# circ_0075943 Dominates the miR-141-3p/AK2 Network to Support the Development of Breast Carcinoma

**DOI:** 10.1155/2021/4098270

**Published:** 2021-11-30

**Authors:** Haixia Wang, Xuechun Zhao, Hui Wang

**Affiliations:** Department of Surgery, Jinan Third People's Hospital, Jinan, Shandong 250101, China

## Abstract

**Background:**

Breast cancer (BC) progression is related to the disorder of circular RNAs (circRNAs). This study aims to characterize the role of circ_0075943 in BC.

**Methods:**

Real-time fluorescent quantitative PCR (real-time PCR) technology was implemented to investigate circ_0075943, AK2 mRNA, and microRNA-141-3p levels. MTT, colony formation method, Transwell, and flow cytometry technique were adopted to investigate cell function. The connection between miR-141-3p and circ_0075943 or AK2 was confirmed by the dual-luciferase reporter gene or RNA immunoprecipitation (RIP). The influence on circ_0075943 in vivo was confirmed by animal experiments.

**Results:**

circ_0075943 was augmented in BC cell lines and tumor specimens. Dwindling of circ_0075943 could dramatically suppress the phenotype of BC cells and induce apoptosis. MiR-141-3p is a target of circ_0075943, and its repression largely reverses the influence of knocking down circ_0075943 on cell behavior. Moreover, AK2, as a target of miR-141-3p, is augmented in BC cells and specimens. AK2 overexpression could restore the phenotype of BC cells blocked by miR-141-3p redevelopment. Moreover, knocking down circ_0075943 could suppress the growth of tumors in vivo.

**Conclusion:**

The abnormal regulation of circ_0075943 participates in part of the expansion of BC by dominating the miR-141-3p/AK2 regulatory network.

## 1. Introduction

Breast cancer is the second incidence of malignant tumors in the world, and the incidence and mortality of breast cancer are consistently ranked first in terms of female-related malignancies [1, 2]. Although the diagnosis and treatment of breast cancer continue to improve with the advancement of medical standards and science and technology, the prognosis of patients with advanced breast cancer is still not optimistic [2, 3]. Deep exploration of the mechanism of breast cancer occurrence and development is still a top priority. With the development of high-throughput sequencing and microarray technology [4], more and more circular RNAs have gradually entered people's field of vision. With the help of bioinformatics analysis, we have a deeper understanding of the biological origin, molecular characteristics, and biological functions of circular RNA.

As a special kind of noncoding RNA, circular RNA (circular RNA, circRNA) connects itself end-to-end to form a circular structure lacking a covalently closed 5′cap and 3′ tail end [5], and we call this different conventional RNA produced by reverse splicing. It is precisely because of this special circular structure where circular RNA has a certain degree of resistance to exonuclease R (RNase), so that circular RNA has a certain degree of stability [6]. Perhaps due to the stability of circular RNA, circular RNA is widespread and abundant in human cells, and the content of circular RNA can even reach more than 10 times its linear isomer. In addition, the expression level of circular RNA in different tissues, organs, cell life processes, and different diseases is also specific [7]. There is growing evidence that circular RNA plays a role in a variety of human diseases [8–12]. The connection between malignant tumors and circular RNA has attracted attention in particular. Studies have confirmed that circular RNA plays an important role in the occurrence and development of breast cancer. circANKS1B can promote the invasion and metastasis of breast cancer in vivo and in vitro by inducing EMT, and the upregulation of its expression is closely related to lymph node metastasis and advanced clinical stage [13]. circTADA2As have the ability to inhibit tumor formation. It is hoped that through the construction of circTADA2As/miR-203a-3p/SOCS3 network targeted therapy for breast cancer is a good prognostic molecular marker for triple-negative breast cancer [14]; other studies have shown that circular RNA also plays an important role in breast cancer resistance; for example, hsa_circ_0025202 in HR-positive breast cancer has an anticancer effect, which can be achieved through the miR-182-5p/FOXO3a axis and can be used as a new marker for tamoxifen resistance [15]. The role of circular RNA in breast cancer is still worthy of continued exploration. The role of circ_0075943 in tumors has not been reported.

As an indicator reflecting the ability of cell metastasis, EMT and MMP families often appear at the same time; there is no exploration on the correlation between EMT and circ_0075943 in breast cancer. In order to enrich the research progress of circ_0075943 in breast cancer, we hope to further explore its internal mechanism.

## 2. Method

### 2.1. Tissue Specimens

Tumor tissue and paracancerous tissue specimens from patients who underwent BC resection in Jinan Third People's Hospital from January 2021 to June 2021 were collected. A total of 30 female patients were enrolled, aged 35–53 years old. Inclusion criteria were as follows: (1) meeting the diagnostic criteria for BC, (2) complete follow-up data and complete medical records for analysis, and (3) no radiotherapy or chemotherapy performed before surgery. Exclusion criteria were as follows: (1) patients with primary malignant tumors in other parts were excluded and (2) clinical data is incomplete and clinical analysis cannot be completed. All patients signed an informed consent form, and this study was reviewed, approved, and supervised by the Jinan Third People's Hospital ethics committee.

### 2.2. Cell Culture and Transfection

Human normal breast epithelial cell lines (MCF-10A) and human BC cell lines (MCF-7, MDA-MB-231, MDA-MB-468, and BT-474) were purchased from Shanghai Cell Biology Research Institute, Chinese Academy of Sciences. MCF-10A, T47D, BT-474, and MDA-MB-468 cells were cultured with RPMI 1640/L-15 medium containing 10% fetal bovine serum. si-NC and si-circ_0075943 and 100 nmol/L of NC, miRNA-141-3p mimics, and miRNA-141-3p inhibitor were infected according to the instructions of LipofectamineTM 3000 transfection reagent. After culturing in a 37°C, 5% CO_2_ incubator for 6 hours, the culture medium was replaced to fresh medium, and the culture was terminated after 48 hours for subsequent experiments.

### 2.3. qPCR Assay

TRIzol reagent was used for extracting total RNA from tissue samples or cells, and a UV spectrophotometer was used to detect the purity and content of total RNA. PrimeScriptTM RT Master Mix kit was used for reversing the extracted circRNA into cDNA. The SYBR Premix EX TaqTM kit was used for qPCR and the FTC-3000p qPCR system for the experiment. 2^−△△Ct^ method was implemented to calculate the relative expression of the target gene. The primer sequences were represented as follows. circ_0075943 upstream primer is 5′-AAGATGAGGGTGTTTACG-3′, downstream primer is 5′-AAGCCTTCTGCCTTAGTT-3′; miR-141-3p upstream primer is 5′-CTCAAGGCAACCTACCGAAAAG-3′, downstream primer is 5′-TATCGGACCCATCACGGAGTGG-3′; AK2 upstream primer is 5′-TTTTCTCTCTACAAGCCATTCCC-3′, the downstream primer is 5′-CCTCCAACTTCCTTGCTTCATCT-3′.

### 2.4. RNA Immunoprecipitation Assay

The extracted cell lysate was incubated with specific antibodies (anti-Ago2/IgG, 1 : 500, Abcam) overnight at 4°C and then pulled down with Protein G-Sepharose beads (Abcam). The beads were washed with lysis buffer five times and then digested with Proteinase K (Sigma‐Aldrich) for 1.5 hours. The digested solution underwent RNA purification using TRIzol Reagent. Quantitative RT-PCR was performed to examine the RNA yield. The primers used for analysis are listed below in the qRT-PCR section.

### 2.5. CCK-8 Assay

BC cells were seeded on a 96-well plate at 2 × 10^4^ cells/well, and when the cells grew to 60% confluence, they were infected with Lipofectamine^TM^ 3000. When incubating for 24, 48, 72, or 96 h, 10 *μ*l CCK-8 solution was added to each well and incubate at 37°C for 2 h. Finally, an automatic microplate reader was used to detect the optical density (OD) value of each well at a wavelength of 450 nm. The experiment was repeated three times.

### 2.6. Transwell Assay

We collect cells after being infected for 48 hours in each group and trypsinize them to make a single cell suspension with a cell density of 1 × 10^6^ cells/ml. We separate the lower and upper chambers of the Transwell cell with a polycarbonate microporous membrane (pore size 8 *μ*m) coated with artificial base glue, add 100 *μ*l cell suspension to the upper chamber, and add 500 *μ*l cell suspension to the lower chamber. Then, we place the Transwell chamber in a cell incubator for 8 hours, take out the polycarbonate microporous membrane, gently wipe the base glue and the cells on the upper surface with a cotton swab, then fix it with neutral formaldehyde, and stain it with hematoxylin. Five fields of view were randomly selected under the microscope, and the number of penetrating cells was counted. The number of penetrating cells represents the cell invasion ability. The experiment was repeated 3 times and the average value was taken.

### 2.7. Cell Migration Test

5 × 10^5^ cells were cultured in a 6-well plate. We scratch the cell surface vertically with the pipette head and washed away the dropped cells with PBS buffer. Then, the cells were cultured at 37° and 5% CO_2_ and photographed by using a microscope (Nikon, Japan) at 0, 6, 12, and 24 hours.

### 2.8. The Effect of circ_0075943 on BC Cell Transplantation Was Observed in Nude Mouse Tumor Model

BC cells were transfected with sh-circ_0075943 and sh-NC. Stable expression BC cell lines were obtained after screening. sh-circ_0075943 BC cells were stabilized and prepared into 1 × 10^7^/mL cell suspension. 200 *μ*L cell suspension was inoculated into the right upper limb armpit of BALB/C nude mice aged 4 weeks. Nude mice were divided into sh-circ_0075943 group and sh-NC group, with 6 mice in each group. The tumor-bearing mice were euthanized by carbon dioxide 28 days after inoculation. We calculate tumor volume (tumor volume = shortest tumor diameter^2^ × longest tumor diameter/2) and weigh its mass.

### 2.9. Histopathology of Tumor in Tumor-Bearing Mice

A part of the tumor fragments was paraffin-embedded, histopathological section, HE staining, and immunohistochemical staining. The histopathological morphology and Ki-67 protein expression were observed under a light microscope. Positive results were quantified by the improved Sinicrope method. Ki-67 protein staining was positive for brownish-yellow particles. A comprehensive score was given according to the proportion of positive cells and staining intensity.

### 2.10. Dual-Luciferase Reporter Geneassay

We insert the predicted circ_0075943 3′UTR or AK2 3′UTR fragment containing miR-145 binding site into pmiRGLO to obtain the reporter gene vector circ_0075943 3′UTR WT and AK2 3′ of wild-type circRNA_001569 3′UTR and AK2 3′UTR UTR WT. The target sequence was subjected to mutation, and the mutation carrier circRNA_001569 3′UTR MUT and AK2 3′UTR MUT were obtained. We cotransfect circ_0075943 3′UTR WT, AK2 3′UTR WT, circ_0075943 3′UTR MUT, or AK2 3′UTR MUT with miRNA-141-3p mimics or NC into BC cells, respectively. After 48 hours, the luciferase activity was measured using a dual-luciferase activity detection kit.

### 2.11. Western Blotting Assay

After 48 hours of transfection, the cells of each group were lysed with RIPA, and total protein was extracted and quantified by the BCA method. The samples were loaded with SDS-PAGE, and the primary antibody was incubated with electrophoresis-transfer-blocking (5% skimmed milk powder) according to the experimental procedure. Rabbit anti-AK2 antibody (1 : 2 000) and rabbit anti-*β*-actin antibody (1 : 2 000), overnight at 4°C, were incubated with secondary antibody, goat anti-rabbit IgG antibody (1 : 1 000), room temperature for 1 h, before development exposure. Image-Pro Plus image analysis system analyzes protein bands.

### 2.12. Statistical Processing

The statistical analysis adopts SPSS 19.0 software, and at least 3 independent experiment results are taken. Normally distributed measurement data are expressed as *x* ± *s*. One-way analysis of variance is adopted for comparison between multiple groups, and LSD-t was adopted for pairwise comparison between multiple groups. The comparison of rates uses the *χ*^2^ test. *P* < 0.05 or *P* < 0.01 indicates that the difference is statistically significant.

## 3. Results

### 3.1. circ_0075943 is Overexpressed in BC Specimen and Cell Lines

**Figure 1:**
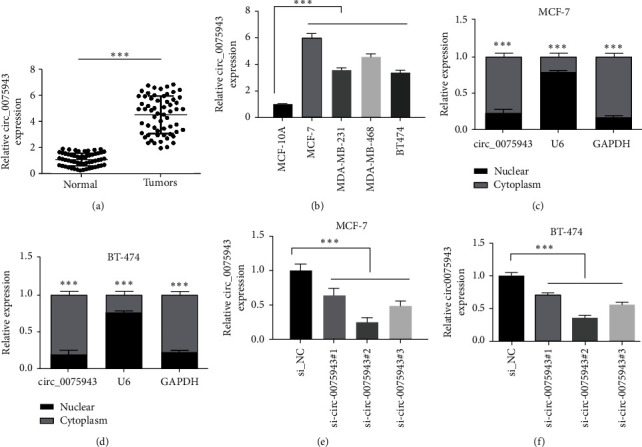
Increased expression of circ_0075943 in BC specimen and cells. (a) Real-time PCR was adopted to check the circ_0075943 level in specimen and normal specimen. (b) Real-time PCR was implemented to check the circ_0075943 level in BC cell lines and noncancerous cell lines (MCF-10A). (c, d) The distribution of circ_0075943 in the nucleus or cytoplasm was studied. (e and f) QRR-PCR was adopted to check the silencing efficiency of circ_0075943. ^*∗∗∗*^*P* < 0.001.

As represented in Figure 1(a), circ_0075943 level in tumor specimen (*n* = 55) was dramatically amplified than that of normal specimen adjacent to cancer (*n* = 55). Moreover, circ_0075943 level in BC cell lines was also dramatically amplified than noncancerous cell lines (MCF-10A) (Figure 1(b)). Further testing exhibited that circ_0075943 is mainly located in the cytoplasm, not in the nucleus (Figures 1(c) and 1(d)). These characteristics indicate that circ_0075943 is abnormally dominated in BC, and circ_0075943 is stably expressed, mainly in the cytoplasm. In order to evaluate the influence on circ_0075943, we adopted siRNA targeting circ_0075943 to restrain expression. The data exhibited that after infection of siRNA-circ_0075943, circ_0075943 level in BC cells was meaningfully diminished, and circ_0075943 level infected with siRNA-circ_0075943#2 was the lowest (Figures 1(e) and 1(f)).

### 3.2. Dwindling of circ_0075943 Intervenes BC Cell Behaviors

**Figure 2:**
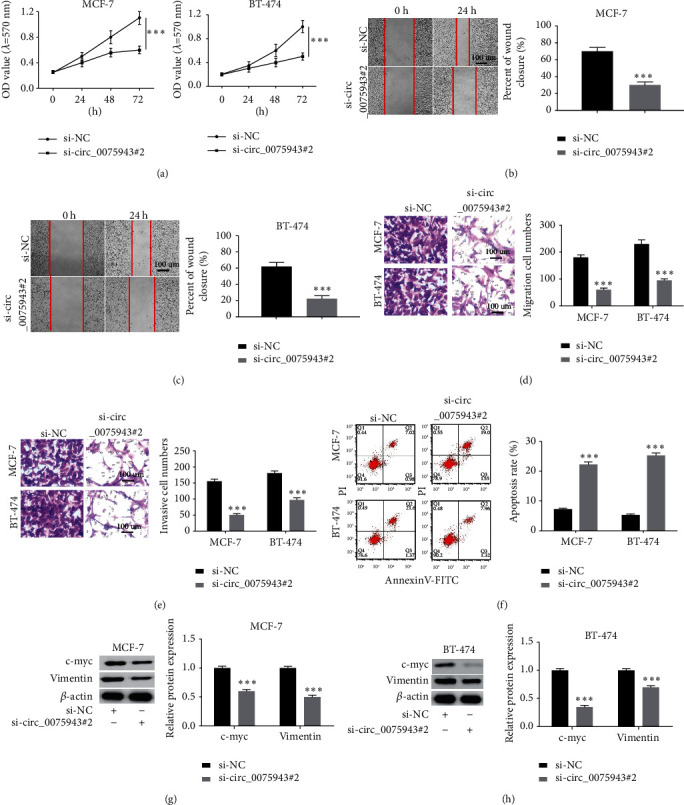
dwindling of circ_0075943 could restrain the phenotype of BC cells and facilitate cell apoptosis and cell cycle arrest. (a) MTT method was adopted to check the influence on exhaustion of circ_0075943 on cell multiplication. (b, c) The influence on circ_0075943 dwindling on cell migration was studied through wound healing experiments. (d, e) The influence on circ_0075943 gene dwindling on cell migration and invasion was evaluated by the Transwell experiment. (f) Flow cytometry was implemented to check the influence on exhaustion of circ_0075943 on cell apoptosis. (g, h) The immunoblotting technique was adopted to check the influence on knocking down circ_0075943 on the expression of c-myc and Vimentin. ^*∗∗∗*^*P* < 0.001.

MTT assay was used for checking cell growth. As represented in Figures 2(a) and 2(b), 72 h after infection, the OD value at 570 nm of the siRNA-circ_0075943 group was dramatically lower than the siRNA-NC group. Wound healing assay and Transwell assay were implemented to check cell migration potential, and Transwell assay was used for checking cell invasion potential. The results exhibited that the wound healing rate and the number of migrating cells of BC cells infected with siRNA-circ_0075943 diminished (Figures 2(b)–2(d)), and the number of invaded cells infected with siRNA-circ_0075943 was also restrained (Figure 2(e)). Flow cytometry exhibited that exhaustion of circ_0075943 could meaningfully facilitate the apoptosis rate of BC cells (Figure 2(f)). Moreover, the data exhibited that after siRNA-circ_0075943 infected BC cells, the levels of c-myc and Vimentin were dramatically restrained (Figures 2(g) and 2(h)). In summary, all data confirm that knocking down circ_0075943 could block the phenotype of BC cells and induce apoptosis.

### 3.3. circ_0075943's Target Protein miR-141-3p Is Downdominated in BC Specimen and Cells

**Figure 3:**
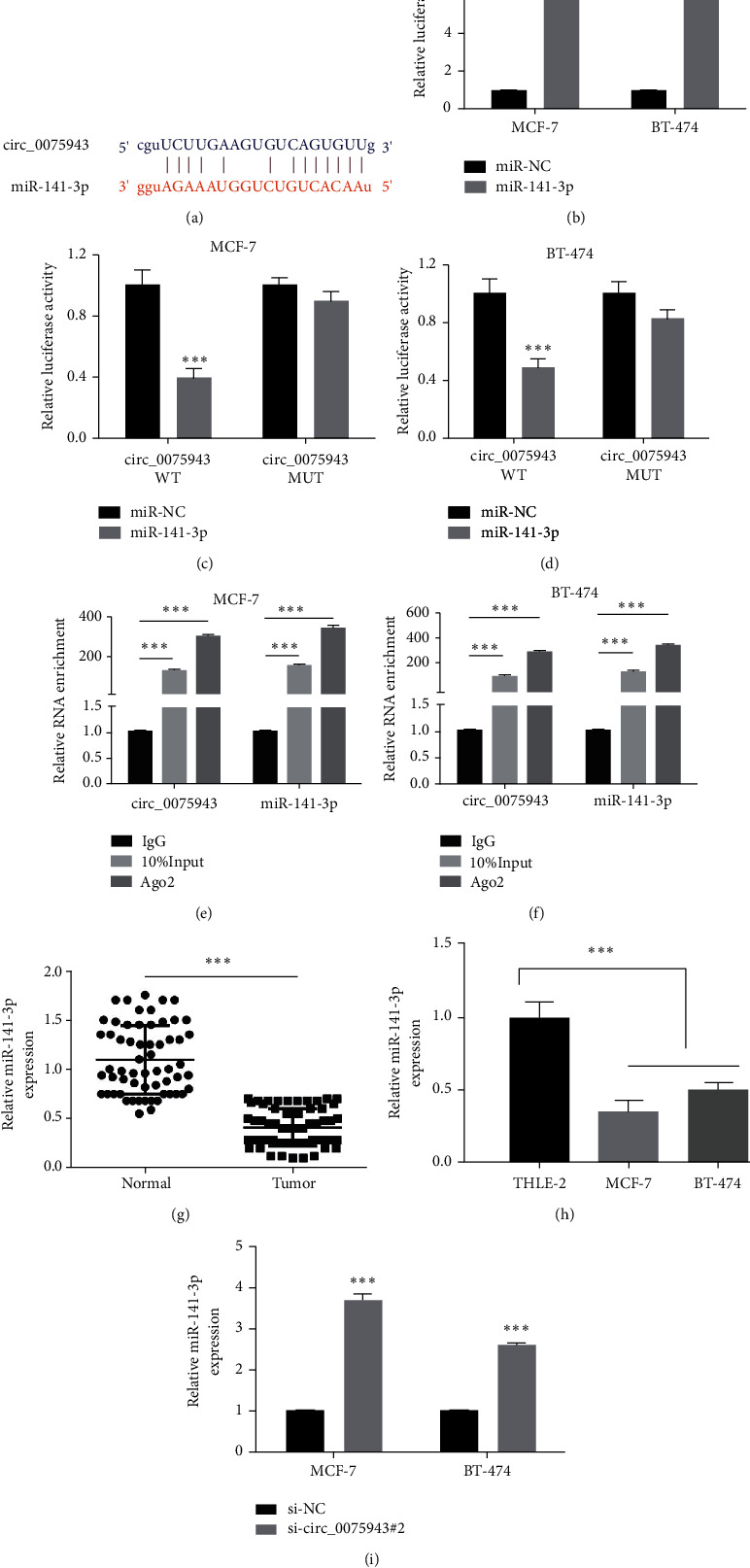
miR-141-3p is the target protein of circ_0075943. (a) The predicted binding site between circ_0075943 and miR-141-3p by Starbase (https://starbase.sysu.edu.cn/). (b) Detection of miR-141-3p mimics efficiency by real-time PCR. (c and d) The interaction between circ_0075943 and miR-141-3p was determined by dual-luciferase reporter gene checked. (e, f) The interaction between circ_0075943 and miR-141-3p was determined by the RIP test. (g) Real-time PCR was adopted to check the miR-141-3p level in tumor specimens and normal specimens. miR-141-3p was low expressed in tumors. (h) Real-time PCR was adopted to check the miR-141-3p level in BC cells. (i) The influence on exhaustion of circ_0075943 gene on the miR-141-3p level was checked by real-time PCR. ^*∗∗∗*^*P* < 0.001.

Through bioinformatics analysis (starBase; http://starbase.sysu.edu.cn/), there are multiple binding sites between circ_0075943 and miR-141-3p (Figure 3(a)), suggesting that miR-141-3p is the potential target of circ_0075943. MiR-141-3p mimics could distinctly enhance the miR-141-3p level in BC cells (Figure 3(b)). Then, dual-luciferase reporter gene detection displayed that the luciferase activity of BC cells infected with miR-141-3p mimic and circ_0075943 WT diminished (Figures 3(c) and 3(d)). Next, the RIP assay displayed that, compared with IgG protein, Ago2 protein enriched circ_0075943 and miR-141-3p (Figures 3(e) and 3(f)). The results displayed that miR-141-3p is one of the target proteins of circ_0075943. Moreover, we observed that miR-141-3p level in tumor specimen (*n* = 55) was distinctly lower than that in adjacent normal specimen (*n* = 55) (Figure 3(g)). Compared with MCF-10A cells, miR-141-3p level in BC cells also diminished (Figure 3(h)). dwindling of circ_0075943 could trigger level of miR-141-3p in BC cells (Figure 3(i)).

### 3.4. circ_0075943 Dominates BC Cell Behavior by Targeting miR-141-3p

**Figure 4:**
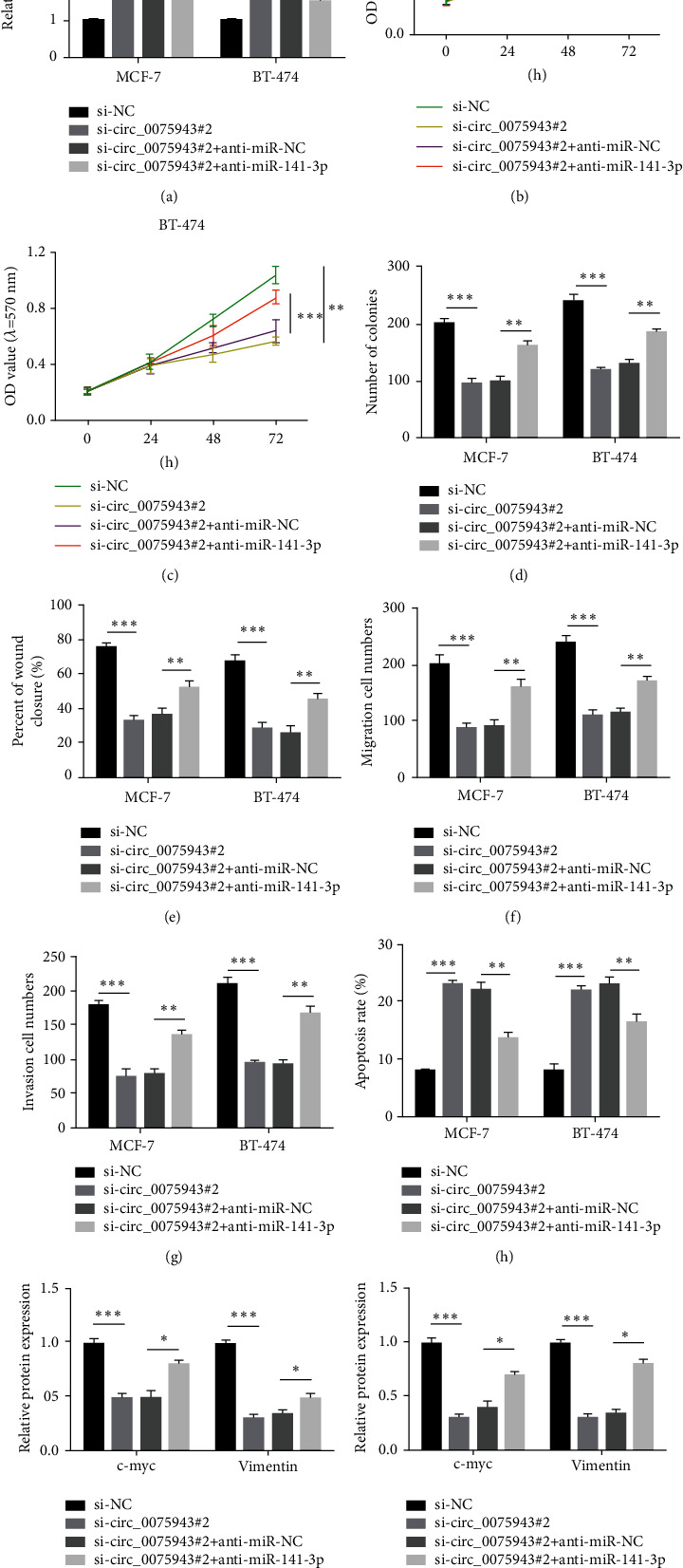
Repression of miR-141-3p reversed the influence on circ_0075943 dwindling. In the rescue experiment, BT-474 and MCF-7 cells were infected with siRNA-circ_0075943 or siRNA-circ_0075943+anti-miR-141-3p, and siRNA-NC or siRNA-circ_0075943+anti-miR-NC was adopted as control. (a) Real-time PCR checks the miR-141-3p level in these cells. (b, c) MTT method was adopted to check cell growth. (d) Detection of cell multiplication by colony formation test. (e and f) Wound healing test and Transwell test were adopted to evaluate cell migration. (g) The Transwell method checks cell invasion. (h) Flow cytometry to check cell apoptosis. (i, j) The immunoblotting technique was adopted to check protein expression. ^*∗*^*P* < 0.05, ^*∗∗*^*P* < 0.01, ^*∗∗∗*^*P* < 0.001.

Next, we investigated whether circ_0075943 plays a role in BC cells by dominating miR-141-3p. In the rescue experiment, BC cells were infected with siRNA-circ_0075943 or siRNA-circ_0075943 + anti-miR-141-3p, and siRNA-NC or siRNA-circ_0075943 + anti-miR-NC were adopted as controls. MiR-141-3p was triggered in cells infected with siRNA-circ_0075943, while the miR-141-3p level was distinctly diminished in cells infected with siRNA-circ_0075943 + anti-miR-141-3p (Figure 4(a)). In BC cells, infection with siRNA-circ_0075943 distinctly restrained cell growing and aggregate formation, but after infection with siRNA-circ_0075943 + anti-miR-141-3p, the cells with proliferation potential and formation potential are partially restored (Figures 4(b)–4(d)). In BC cells, exhaustion of circ_0075943 alone restrained cell migration and invasion capabilities, while combined suppression of miR-141-3p greatly facilitated cell migration and invasion capabilities (Figures 4(e)–4(g)). Additional suppression of miR-141-3p could restrain the apoptosis induced by circ_0075943 dwindling (Figure 4(h)). Moreover, the protein levels of c-myc and Vimentin in BC cells infected with siRNA-circ_0075943 diminished, but in cells infected with siRNA-circ_0075943 + anti-miR-141-3p, c-myc and Vimentin recovered distinctly (Figures 4(i) and 4(j)). Experimental outcomes show that knocking down circ_0075943 could restrain the malignant behavior of BC cells by elevating miR-141-3p level.

### 3.5. circ_0075943 Dominates AK2 Level by Targeting miR-141-3p

**Figure 5:**
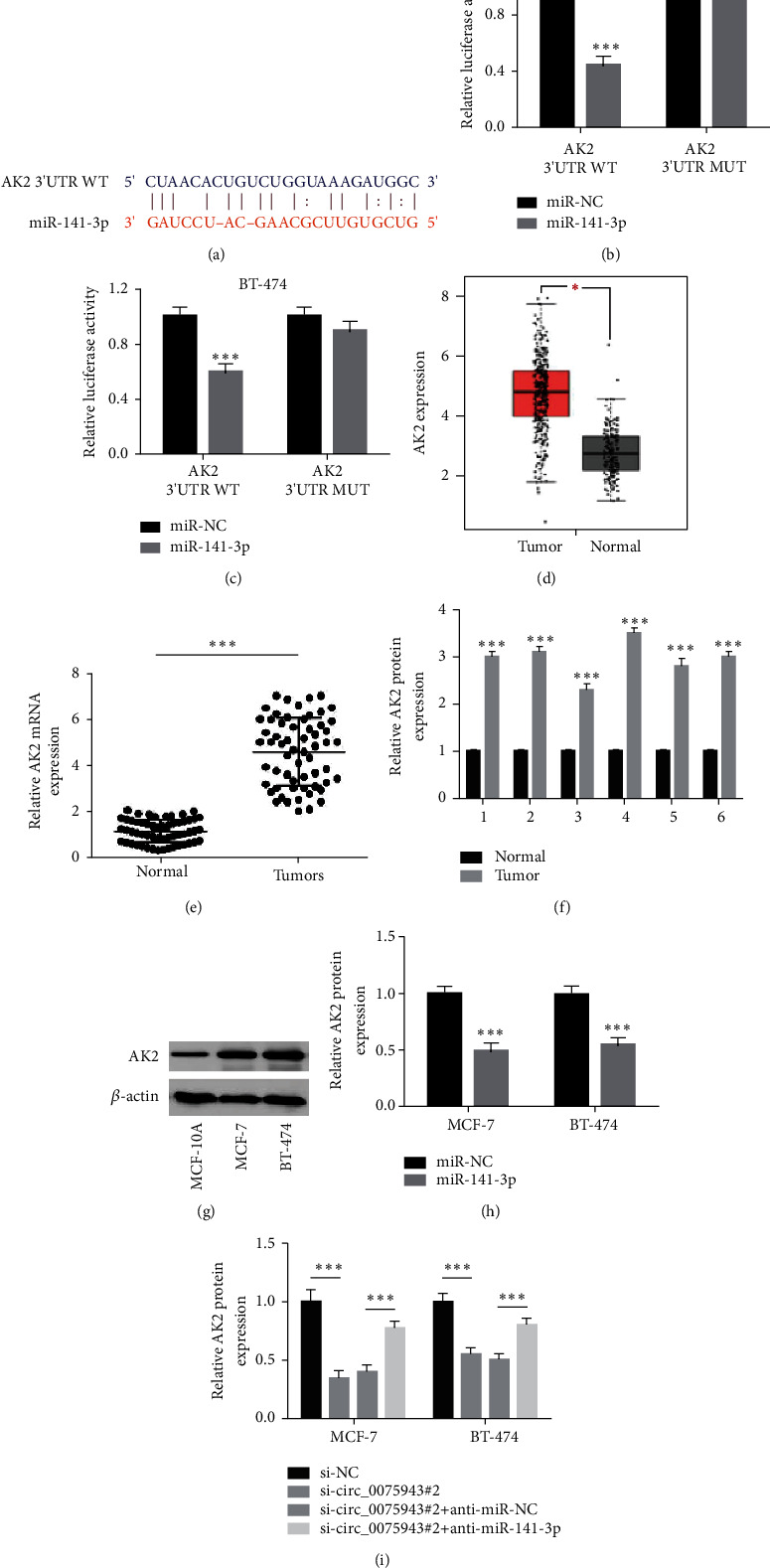
circ_0075943 dominates AK2 level by targeting miR-141-3p. (a) The binding site of AK2 3′UTR and miR-141-3p is provided by TargetScan (http://www.targetscan.org/vert_71/). (b, c) The interaction between AK2 and miR-141-3p was affirmed by the dual-luciferase reporter gene. (d) AK2 level in the normal specimen and BC tumor specimen from the GEPIA database (http://gepia.cancer-pku.cn/). (e, f) Detect AK2 level in our tissue samples by real-time PCR and immunoblotting technique. (g) The immunoblotting technique was adopted to check AK2 protein level in MCF-10A, BC cells. (h) The immunoblotting technique was adopted to check the influence on miR-141-3p redevelopment on AK2 level protein. (i) Immunoblotting technique checked AK2 protein in BC cells. ^*∗∗∗*^*P* < 0.001.

Through bioinformatics analysis, there is a binding site between miR-141-3p and AK2 3′UTR (Figure 5(a)), indicating that AK2 may be a miR-141-3p target. This prediction was affirmed by a luciferase reporter gene test. The results displayed that after coinfection of miR-141-3p mimic and AK2 3′utr WT, the luciferase activity in BC cells was significantly decreased (Figures 5(b) and 5(c)). GEPIA data displayed that, compared with normal samples, AK2 level was distinctly triggered (Figure 5(d)). Similarly, AK2 mRNA and protein levels in tumor specimens (*n* = 55) were also distinctly higher than those in normal specimens adjacent to cancer (*n* = 55) (Figures 5(e) and 5(f)). Compared with MCF-10A cells, AK2 level protein in BC cells is also triggered (Figure 5(g)). Moreover, we observed that miR-141-3p overexpression distinctly dwindled AK2 level protein (Figure 5(h)). Interestingly, compared with siRNA-NC, AK2 level in BC cells infected with siRNA-circ_0075943 was distinctly diminished but compared with siRNA-circ_0075943 + anti-miR-141-3p. Compared with the cells infected with siRNA-circ_0075943 + anti-miR-NC, the AK2 level was largely restored (Figure 5(i)). It is suggested that circ_0075943 indirectly dominates the AK2 level by targeting miR-141-3p.

### 3.6. The Restoration of miR-141-3p Inhibits BC Cell by Inhibiting AK2 and Facilitates Cell Apoptosis

**Figure 6:**
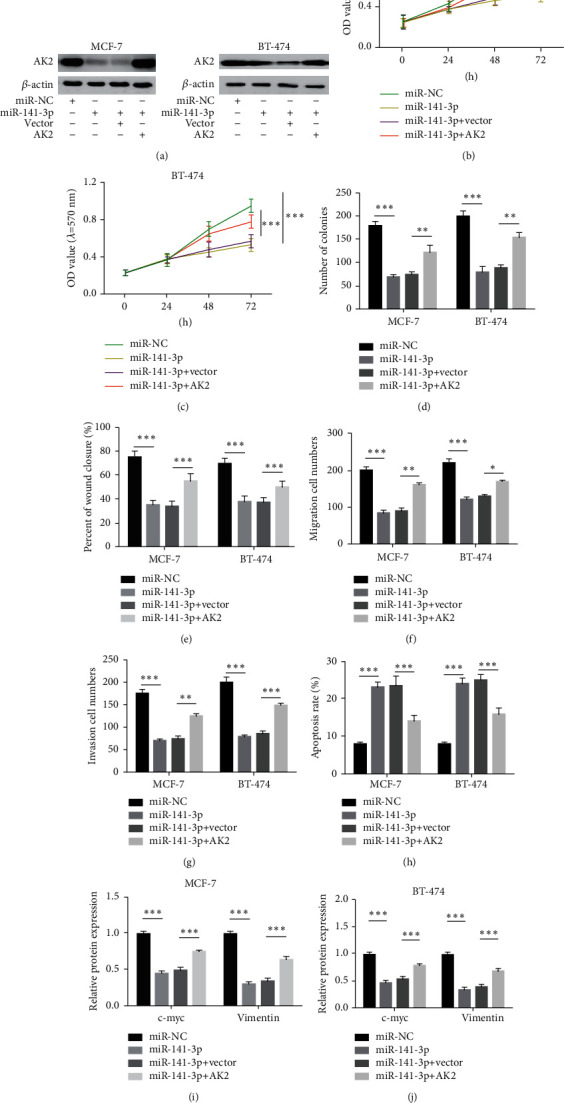
Recovery of miR-141-3p suppresses BC cell phenotype by degrading AK2. Transfect miR-141-3p or miR-141-3p + AK2 in BC cells, and conduct rescue experiments with miR-NC or miR-141-3p + vector as a control. (a) The immunoblotting technique checks AK2 level protein in MCF-7 and BT-474 cells. (b, c) MTT method was adopted to check cell growth. (d) The colony formation test observes cell multiplication. (e, f) Evaluation of cell migration by Transwell and wound healing test. (g) The Transwell experiment evaluates cell invasion potential. (h) Flow cytometry to check cell apoptosis. (i, j) Flow cytometry to monitor cell cycle progression. (k, l) The immunoblotting technique checked Vimentin and c-myc protein levels. ^*∗*^*P* < 0.05, ^*∗∗*^*P* < 0.01, ^*∗∗∗*^*P* < 0.001.

Next, we investigated whether miR-141-3p works by suppressing AK2 levels. Compared with miR-NC, the AK2 level in the cells infected with miR-141-3p was distinctly restrained, and compared with the miR-141-3p + vector, AK2 level in the cells infected with miR-141-3p + AK2 was largely restored (Figure 6(a)). The restoration of miR-141-3p dramatically suppressed cell growth and colony formation potential, and the introduction of AK2 could induce cell multiplication and colony formation (Figures 6(b)–6(d)). In BC cells, the overexpression of miR-141-3p distinctly blocked the cell migration and invasion potential, while the addition of AK2 restored the cell migration and invasion potential to a large extent (Figures 6(e)–6(g)). Infection of miR-141-3p + AK2 could restrain the apoptosis of BC cells, and infection of miR-141-3p alone could distinctly accelerate cell apoptosis (Figure 6(h)). Moreover, in BC cells, the protein levels of miR-141-3p infected with c-myc and Vimentin were mainly restrained by miR-141-3p + AK2 infection (Figures6(k) and 6(l)). These results indicate that miR-141-3p restrains the malignant behavior of BC cells by restraining AK2.

### 3.7. circ_0075943 Dwindling Suppressed Tumor Growth In Vivo

**Figure 7:**
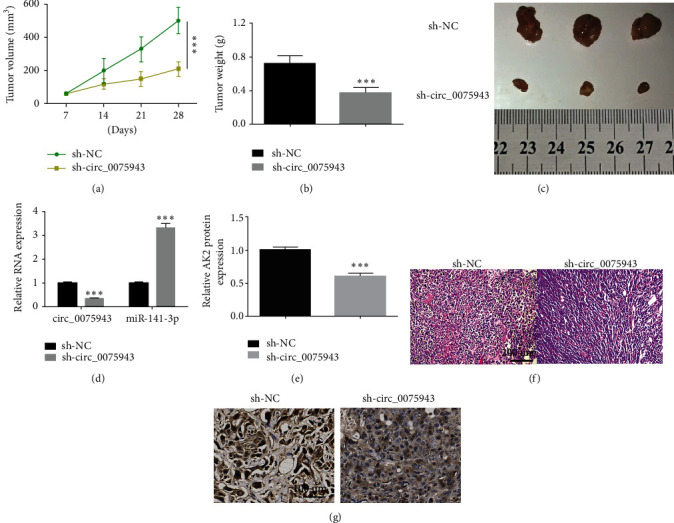
Exhaustion of circ_0075943 restrained tumor growth in vivo. (a) The tumor volume is measured once a week to assess tumor growth. (b) Measure tumor weight to assess tumor growth. (c) Representative image of tumor tissue. (d) miR-141 level-3p and circ_0075943 in tumor specimen were checked by real-time PCR. (e) The immunoblotting technique checks the AK2 level in the tumor specimen. Silence circ_0075943 reduces AK2 expression. (f) Histological analysis of different groups of the specimen by HE staining. (g) The immunohistochemical method was adopted to check Ki-67 in tumor specimens of each group. Silencing circ_0075943 reduced the proliferation ability of BC cells. ^*∗∗∗*^*P* < 0.001.

We further confirmed the role of circ_0075943 in BC through animal experiments. Compared with sh-NC-infected BT-474 cells, sh-circ_0075943-infected BT-47 cells had slower tumor growth and restrained tumor volume (Figure 7(a)). We measured the tumor weight of each tissue. The data exhibited that circ_0075943 dwindling dramatically restrained the tumor weight, resulting in smaller tumor size (Figures 7(b) and 7(c)). Moreover, we observed that circ_0075943 level in the tumor specimen of the sh-circ_0075943 experimental group diminished, while the miR-141-3p level was triggered (Figure 7(d)). The AK2 protein level in the tumor specimen of the sh-circ_0075943 experimental group was meaningfully restrained (Figure 7(e)). Moreover, HE staining was performed on the tissue morphology, and it was observed that circ_0075943 dwindling slowed the development of tumors (Figure 7(f)). Moreover, immunohistochemical staining exhibited that Ki-67 in the sh-circ_0075943 experimental group was meaningfully restrained (Figure 7(g)). All data indicated that knocking down circ_0075943 restrained tumor growth in vivo.

## 4. Discussion

BC is the leading cause of cancer-related deaths in women worldwide [16, 17], the most common cancer in women in the United States (except skin cancer) and the second leading cause of cancer deaths in women after lung cancer [18]. Therefore, it is very important to clarify the mechanisms related to the occurrence and development of BC and find promising therapeutic earmarks for BC treatment.

circRNA is a new type of endogenous RNA that is highly expressed in the eukaryotic transcriptome. Compared with lncRNA and miRNA, circRNA has a covalently closed continuous loop instead of a 5′-3′ polar and polyadenylated tail [19]. Due to this special stable structure, circRNA can participate in various biological functions, thereby improving biological stability. This study observed that circ_0075943 is highly expressed in BC tissues and cells, and knocking down circ_0075943 can inhibit BC cell malignancy. Therefore, circ_0075943 may be adopted as a new type of potential biomarker or cancer treatment earmark.

MiRNAs constitute a class of small single-stranded noncoding RNA, and thousands of miRNAs are believed to be involved in metabolism, signal transduction, cell adhesion, cell motility, cell differentiation, and apoptosis [20–22]. Therefore, for different earmark genes and cancer types, miRNAs have been observed to be both oncogenes and tumor suppressors [23]. MiRNA-141 is one of the miR-200 family members. Studies [24, 25] confirmed that miR-141 has abnormal expression in a variety of malignant tumors, including BC, colorectal cancer, bladder cancer, and renal clear cell carcinoma, suggesting that miR-141 may be involved in the development of tumors. A recent study [26] observed that miR-141 can endorse the multiplication of ovarian cancer cells by inhibiting SIK1. MiR-141 level in gastric cancer tissues is significantly reduced and suggests a poor prognosis [27]. This study observed that overexpression of miR-141-3p can restrain BC cell malignancy and endorse BC cell apoptosis. Moreover, with the help of bioinformatics tools to predict the binding site of circ_0075943 and miR-141-3p, dual-luciferase reporter gene detection further confirmed the targeting relationship between circ_0075943 and miR-141-3p. At the same time, it was observed that circ_0075943 was negatively correlated with miR-141 level-3p.

Adenylate kinase (AK) family isoenzymes are the machinery of nucleotide synthesis in cells. The nucleotide is not only a component of the nucleic acid structure but also acts as a source of chemical energy or as an activation intermediate in cell metabolism, many biosynthetic pathways, and cell signal transduction or as a component of a coenzyme. AK2 is the main adenylate kinase subtype located in the inner mitochondrial membrane, and its gene is located at 1p34 [28]. Northern blot analysis showed that AK2 mRNA was higher in the liver, heart, skeletal muscle, and pancreas and slightly less in the kidney, placenta, and brain [29, 30]. This study observed that AK2 is highly expressed in BC tissues and BC cells, and overexpression of AK2 endorses the malignancy of BC cells. Using bioinformatics tools to predict the binding site of miR-141-3p and AK2, dual-luciferase reporter gene detection further confirmed the targeting relationship of miR-141-3p and AK2 and observed that miR-141-3p and AK2 expression is negatively correlated. AK2 might be a target for the treatment of breast cancer.

In summary, miR-141-3p earmarks negative regulation of circ_0075943 and AK2, and circ_0075943/miR-141-3p/AK2 axis can endorse the malignancy of BC cells, and they may be potential earmarks for BC treatment.

## Data Availability

The data used to support the findings of this study are available from the corresponding author upon request.
